# Effects of replacing soybean meal with an equivalent amount of fermented feed on the production performance and egg and meat quality of laying hens during the late laying period

**DOI:** 10.5194/aab-68-445-2025

**Published:** 2025-07-04

**Authors:** Zhongwei Miao, Linli Zhang, Li Li, Jianfeng Pei, Shenyi Shi, Zhiming Zhu, Qinlou Huang, Nenzhu Zheng, Qingwu Xin

**Affiliations:** 1 Institute of Animal Husbandry and Veterinary Medicine, Fujian Academy of Agricultural Sciences/Fujian Key Laboratory of Animal Genetics and Breeding, Fuzhou, 350013, China; 2 Fujian Bai yuan hang Agricultural Development Company, Ltd., Sanming, 365000, China

## Abstract

This study aimed to investigate the effects of replacing soybean meal with an equivalent amount of fermented feed on the production performance of laying hens. A total of 480 healthy, 70-week-old Hy-Line Sonia laying hens were divided into three groups, with four replicates of 40 chickens in each group. The control group (NT) was fed a basal diet, while the experimental groups were fed with a diet in which soybean meal was replaced with an equivalent amount of fermented feed: 5 % replacement in treatment group 1 (TT1) and 10 % replacement in treatment group 2 (TT2). At 8 weeks after soybean meal replacement, the results showed that the abnormal egg rate in the 10 % fermented feed group was significantly reduced. Egg quality parameters, such as eggshell strength and thickness, significantly improved in both the TT1 and TT2 groups after 4 and 8 weeks of equivalent soybean meal replacement. The drip loss of breast muscle was significantly lower in the TT1 and TT2 groups compared to the NT group at both 4 and 8 weeks. In addition, there was a significant increase in the pH of thigh muscle at 0 h postmortem and a significant reduction in the fat and cholesterol contents in the muscles of laying hens in the TT1 and TT2 groups. Replacement of 10 % soybean meal with an equivalent amount of fermented feed for 8 weeks yielded a satisfactory effect.

## 1 Introduction

To increase food safety and reduce the harm caused by the misuse of antibiotics (Chen and Yu, 2022), the use of antibiotics has been completely banned in China's feed industry since 2020. In recent years, international trade friction has led to record-high prices for corn and soybean meal in raw feed materials, causing the feed cost for laying hens to continuously increase. This has required the layer farming industry to further improve the feed nutrition and health of laying hens and adjust the nutritional design of feed. Fermented feed, as a new type of feed additive, has promising application prospects and can improve feed palatability and utilization (Missotten et al., 2015), thereby enhancing animal production performance, reducing enterprise production costs, and increasing economic benefits.

Fermented feed is a type of biological feed produced by fermenting plant-based agricultural byproducts as raw materials through the exogenous addition of beneficial microorganisms (Teran et al., 2022). Studies have shown that fermented feed is rich in nutrients and produces a variety of digestive enzymes; moreover, fermentation can reduce the content of antinutrients, increase the nutritional value and utilization of feed, and improve animal production performance (Ashayerizadeh et al., 2017; Li et al., 2019; Jazi et al., 2019; Chang and Yu, 2022; Wang et al., 2023). Fermented feed has been shown to improve the laying performance, egg quality, meat quality, muscle fatty-acid content lipid metabolism, intestinal flora, and nitrogen and phosphorus excretion of chickens to varying degrees (Xu et al., 2012; Qaisrani et al., 2015; Yu et al., 2018; Krauze et al., 2021;Guo et al., 2021; Liu et al., 2022; Darmawan et al., 2022). With continuous improvements in the production performance of specialized laying hens, egg quality and meat quality have gradually declined; however, national dietary habits indicate increasingly high requirements for egg quality and meat quality. Therefore, various types of fermented feed additives that can be used to fully exploit the production potential of laying hens while also improving quality are increasingly favored when considering feed for laying hens (LutfulKabir, 2009).

The number of laying hens farmed in China has consistently ranked first in the world. In 2022, China had approximately 1 billion laying hens, with egg production of approximately $18.84\times 10^{6}$ t (Yang, 2021). Chicken consumption in China is second only to pork consumption. In 2022, China's chicken consumption was $14.6\times 10^{6}$ t, of which meat production from late-laying (culled) hens (mostly 72–80 weeks old) accounted for approximately 13.2 % of the total chicken production (Liu et al., 2013). China has a vast number of late-laying (culled) hens, and the corresponding income is an important part of the total revenue from layer farming. However, the current selling price of late-laying (culled) hens is generally low. Research has shown that the meat of late-laying (culled) hens is characterized by high protein, low fat, and low cholesterol levels; however, their muscle has a significantly lower water-holding capacity than that of broiler chickens (El-Tarabany et al., 2022). Studies on late-laying (culled) hens have focused mostly on processing, improvements, and utilization after slaughter; there are few studies on improvements before slaughter. Therefore, in this study, 70-week-old Hy-Line Sonia laying hens were used as research objects to investigate the effects of the equivalent replacement of soybean meal with fermented feed on the egg quality, meat quality, and muscle nutritional composition of late-laying hens, with the goal of providing a theoretical basis for the widespread application of fermented feed in the production of late-laying hens.

## 2 Materials and methods

### 2.1 Experimental animals and materials

A total of 480 healthy, 70-week-old Hy-Line Sonia laying hens with similar production performance and nearly the same weights were randomly selected and divided into a control group (NT), a 5 % replacement group (TT1), and a 10 % replacement group (TT2), with four replicates per group and 40 chickens per replicate. The NT group was fed a basal diet, whereas 5 % and 10 % of the soybean meal in the basic diet was replaced with an equal quantity of fermented feed in the respective TT1 and TT2 groups. The experiment consisted of a 2-week trial period and an 8-week formal period. Experimental data were collected for analysis at the end of the fourth and eighth weeks of the formal period. The basal diet was formulated in-house; its components and nutritional composition are shown in Table 1. The fermented feed was provided by Fujian Bai yuan hang Agricultural Development Co., Ltd. The basal diet and fermented feed were sent to Xiamen Pony Testing Co., Ltd. for analyses of nutritional levels. The nutritional compositions of the diets are presented in Table 1.

**Table 1:** Composition and nutrient level of the basal diet (on a dry-matter basis) and dry fermented feed.

Ingredients	Content (%)	Estimated nutritional value^3^	Content (%)	
			Basal diet	Dry fermented feed
Corn	61	ME^2^/(MJ kg^−1^)	10.71	11.22
Soybean meal	25	CP	16.45	28.9
Limestone	9	Ca	4.28	2.31
Premix^1^	5	TP	0.65	8.68
		NaCl	0.42	0.33
		Crude fiber	3.5	12.2
		Ash	11.7	13.1
		Fat	3.2	2.3
		Moisture	9.6	23.6

^1^ The premix provided the following per kilogram of the feed: vitamin A, 8500 IU (international unit); vitamin D, 3000 IU; vitamin E, 25 IU; vitamin B, 12 mg; vitamin K, 2.4 mg; Mn, 90 mg; Zn, 70 mg; Fe, 20 mg; Cu, 10 mg; Co, 0.1 mg; and Se, 0.3 mg. ^2^ All values are measured values. ^3^ Abbreviations in the column are as follows: ME – metabolizable energy; CP – crude protein; TP – total phosphorus.

### 2.2 Feeding management

The experiments were carried out at the Poultry Experimental Base of the Institute of Animal Husbandry and Veterinary Medicine at the Fujian Academy of Agricultural Sciences. The chickens were raised in a three-level, ladder-type, A-type cage system, with individual cages at each level. There were 40 cages per replicate, with a gap of 5 cages between replicates. Throughout the experiment, the chickens were fed powdered feed and provided free access to water. The feed intake was adjusted to 120 g per chicken per day, divided into three feedings per day (06:00, 11:00, 18:00 LT), with egg collection at 09:00 LT daily. The indoor temperature was maintained at 20–25 °C, with a light intensity of 25 lx and a daily light duration of 16 h.

### 2.3 Production performance

During the formal experimental period, the number of eggs laid, the number of abnormal eggs, and the number of dead and culled laying hens in each group were recorded every day, with each replicate as a unit. Using these data, the egg-laying rate, abnormal egg rate, and mortality and culling rates were calculated for each group.

### 2.4 Determination of egg quality

At the end of the fourth and eighth weeks of the experiment, five eggs were randomly selected from each replicate ( n= 20). The egg weight, yolk weight, and eggshell weight were accurately measured using an electronic balance (Ohaus CP1502), and the yolk ratio was calculated. The eggshell weight was determined after the egg contents were removed. The longitudinal and transverse diameters of the eggs were measured using a digital Vernier caliper (SL-102), and the egg shape index was calculated. Eggshell strength was measured using an eggshell strength tester (Tianxiang KQ-1A). Yolk color, albumen height, and haugh units were measured using an egg quality analyzer (Orka EA-01). Eggshell thickness was measured at the blunt, middle, and sharp ends using a digital thickness gauge (SL-325), and the average of the three measurements was calculated.

### 2.5 Determination of meat quality and muscle nutritional composition

Five chickens were randomly selected from each replicate and euthanized via cervical dislocation. The animals were maintained and processed in accordance with the guidelines and regulations of the Animal Care and Use Ethics Committee of the Institute of Animal Husbandry and Veterinary Research of the Fujian Academy of Agricultural Sciences (approval no. 202402FJ001). The breast and thigh muscles from the left and right sides were sampled to determine meat quality according to “NY/T 1333-2007 Determination of livestock and poultry meat quality” (Ministry of Agriculture and Rural Affairs of the People’s Republic of China, 2007). Using a meat quality pH meter (Testo 205), the pH values of three points in the breast and thigh muscles were measured, and the average was calculated. The color of the breast muscle was determined via an NH310 colorimeter. Muscle drip loss was measured over different time intervals using a drip loss measuring cup. Samples of breast muscle were weighed, cooked at 75 °C for 1 h, and cooled at room temperature for 20 min. After excess water was absorbed from the surface, the samples were weighed again, after which the degree of cooking loss was calculated. The shear force of the meat samples was measured using a muscle tenderness instrument (Bulader TS-100). In accordance with the GB5009–2016 guidelines (National Health and Family Planning Commission, State Food and Drug Administration, 2016), moisture content was measured using the direct drying method, ash content was measured using the ignition method, crude protein content was measured using the Kjeldahl method, crude fat content was measured using Soxhlet extraction, cholesterol content was measured using high-performance liquid chromatography, amino acid content was measured using an automatic amino acid analyzer, and fatty-acid content was measured using gas chromatography. The selenium content in the muscle was measured using fluorescence spectrophotometry (GB 5009.93-2017; National Health and Family Planning Commission, State Food and Drug Administration, 2017).

### 2.6 Data statistics and analysis

The experimental data were statistically analyzed using the SPSS 19.0 software. Significance testing was performed using one-way analysis of variance (ANOVA) with the least significant difference (LSD) test and Tukey's multiple comparison test, with P < 0.05 indicating a significant difference and P < 0.01 indicating a highly significant difference. The data are expressed as the means ± standard deviations (SDs).

## 3 Results

### 3.1 Effects of the replacement of different amounts of soybean meal with equivalent amounts of fermented feed on the production performance and egg quality

As shown in Table 2, 4 weeks after the replacement of soybean meal with an equivalent amount of fermented feed, the egg-laying rate, abnormal egg rate, and culling rate in the TT1 and TT2 groups were not significantly different from those in the NT group ( P > 0.05). At 8 weeks after replacement, the egg-laying rates and mortality and culling rates in the TT1 and TT2 groups were not significantly different from those in the NT group ( P > 0.05), but the abnormal egg rate in the TT2 group was significantly lower than that in the NT group ( P < 0.01).

**Table 2:** Effects of the replacement of soybean meal with equivalent amounts of fermented feed on the laying performance.

Items	Time	Dry meal addition level of fermented feed			P value
		NT	TT1	TT2	
Laying rate (%)	Week 4	75.39 ± 3.7	75.57 ± 4.17	76.33 ± 2.03	0.979
	Week 8	84.11 ± 0.99	83.231 ± 0.93	84.49 ± 0.92	0.364
Abnormal egg rate (%)	Week 4	2.59 ± 0.25	2.54 ± 0.32	2.33 ± 0.35	0.484
	Week 8	1.60 ± 0.16^A^	1.50 ± 0.20^A^	0.80 ± 0.18^B^	0.006
Mortality (%)	Week 4	0.83	0.83	0.83	
	Week 8	0	0.83	0.83	

Note that values with different uppercase superscripts in the same row denote an extremely significant difference ( P<0.01 ), values with different lowercase superscripts in the same row denote a significant difference ( P<0.05 ), and values with the same letter superscript or no letter in the same row denote no significant difference ( P>0.05 ).

**Table 3:** Effects of the replacement of soybean meal with equivalent amounts of fermented feed on the egg quality.

Items	Time	Dry meal addition level of fermented feed			P value
		NT	TT1	TT2	
Egg weight (g^−1^)	Week 4	60.44 ± 4.40	59.20 ± 4.24	60.12 ± 4.44	0.228
	Week 8	60.94 ± 4.43^Bb^	61.07 ± 3.92^ABb^	63.30 ± 4.16^Aa^	0.005
Egg yolk ratio (%)	Week 4	26.17 ± 1.69	26.15 ± 4.21	26.95 ± 1.91	0.099
	Week 8	27.11 ± 2.01	26.84 ± 1.47	26.82 ± 1.51	0.598
Shell weight (g^−1^)	Week 4	6.53 ± 0.66	6.58 ± 0.57	6.63 ± 0.67	0.604
	Week 8	6.61 ± 0.56^B^	6.76 ± 0.56^B^	7.10 ± 0.55^A^	0.000
Egg shape index	Week 4	1.31 ± 0.05	1.29 ± 0.10	1.31 ± 0.04	0.245
	Week 8	1.31 ± 0.04	1.31 ± 0.04	1.31 ± 0.04	0.936
Yolk color	Week 4	5.35 ± 0.79^b^	5.64 ± 0.94^ab^	5.93 ± 1.08^a^	0.019
	Week 8	5.03 ± 0.97	5.17 ± 0.87	5.34 ± 1.05	0.239
Eggshell strength (kg cm^−2^)	Week 4	4.75 ± 1.14^b^	5.25 ± 1.33^a^	5.38 ± 1.24^a^	0.023
	Week 8	4.74 ± 0.99^Bb^	5.11 ± 1.02^ABb^	5.62 ± 0.92^Aa^	0.000
Haugh unit	Week 4	69.50 ± 7.33	68.60 ± 5.81	67.76 ± 8.69	0.565
	Week 8	73.51 ± 8.40	72.53 ± 5.95	72.48 ± 6.02	0.356
Albumen height (mm)	Week 4	5.27 ± 0.97	5.07 ± 0.71	5.01 ± 1.04	0.447
	Week 8	5.76 ± 1.17	5.63 ± 0.80	5.63 ± 0.82	0.686
Eggshell thickness (mm)	Week 4	0.39 ± 0.03^b^	0.40 ± 0.07^a^	0.40 ± 0.04^a^	0.048
	Week 8	0.39 ± 0.03^Bb^	0.40 ± 0.02^ABa^	0.41 ± 0.03^A^	0.001

See the footnote of Table 2 for an explanation of the use of subscript letters in the table.

As shown in Table 3, at 4 weeks after the replacement of soybean meal with an equivalent amount of fermented feed, both eggshell strength and thickness significantly improved in the TT1 group ( P < 0.05); the other egg quality indicators did not change significantly ( P > 0.05). In the TT2 group, yolk color, eggshell strength, and eggshell thickness significantly improved ( P < 0.05); the other egg quality indicators did not change significantly ( P > 0.05). At 8 weeks after replacement, compared with the NT group, the TT2 group presented significant increases in egg weight, eggshell weight, eggshell strength, and eggshell thickness ( P < 0.01), whereas the TT1 group presented significant increases in eggshell thickness ( P < 0.05), with no notable differences observed in other egg quality indicators ( P > 0.05).

### 3.2 Effects of the replacement of different amounts of soybean meal with equivalent amounts fermented feed on the meat quality of laying hens

As shown in Table 4, at 4 and 8 weeks after the replacement of soybean meal with an equivalent amount of fermented feed, the shear force and cooking loss of breast muscle in the TT1 and TT2 groups did not differ significantly from those in the NT group ( P > 0.05); however, the drip loss of breast muscle (at 24 and 48 h) was significantly lower in the TT1 and TT2 groups compared with the NT group ( P < 0.05 or P < 0.01).

**Table 4:** Effects of the replacement of soybean meal with equivalent amounts of fermented feed on the breast muscle quality.

Items	Time	Dry meal addition level of fermented feed			P value
		NT	TT1	TT2	
Shear force (kgf)^*^	Week 4	3.80 ± 1.29	3.38 ± 0.82	4.67 ± 0.80	0.195
	Week 8	4.53 ± 2.57	3.25 ± 1.01	2.29 ± 0.83	0.159
Cooking loss (%)	Week 4	8.89 ± 2.39	8.27 ± 2.69	9.64 ± 0.80	0.385
	Week 8	7.84 ± 0.55	9.58 ± 2.69	8.53 ± 1.42	0.143
Drip loss (%) (24 h)	Week 4	9.73 ± 1.63^Aa^	5.99 ± 1.20^ABb^	3.45 ± 1.06^Bb^	0.004
Drip loss (%) (48 h)		19.15 ± 3.82^a^	10.18 ± 0.58^b^	9.44 ± 2.39^b^	0.028
Drip loss (%) (24 h)	Week 8	4.94 ± 0.37^Aa^	3.47 ± 0.54^ABb^	2.72 ± 0.92^Bb^	0.001
Drip loss (%) (48 h)		7.17 ± 0.58^a^	5.29 ± 0.47^b^	5.33 ± 0.50^b^	0.021

^*^ The unit “kgf” denotes kilogram force. See the footnote of Table 2 for an explanation of the use of subscript letters in the table.

**Figure 1:**
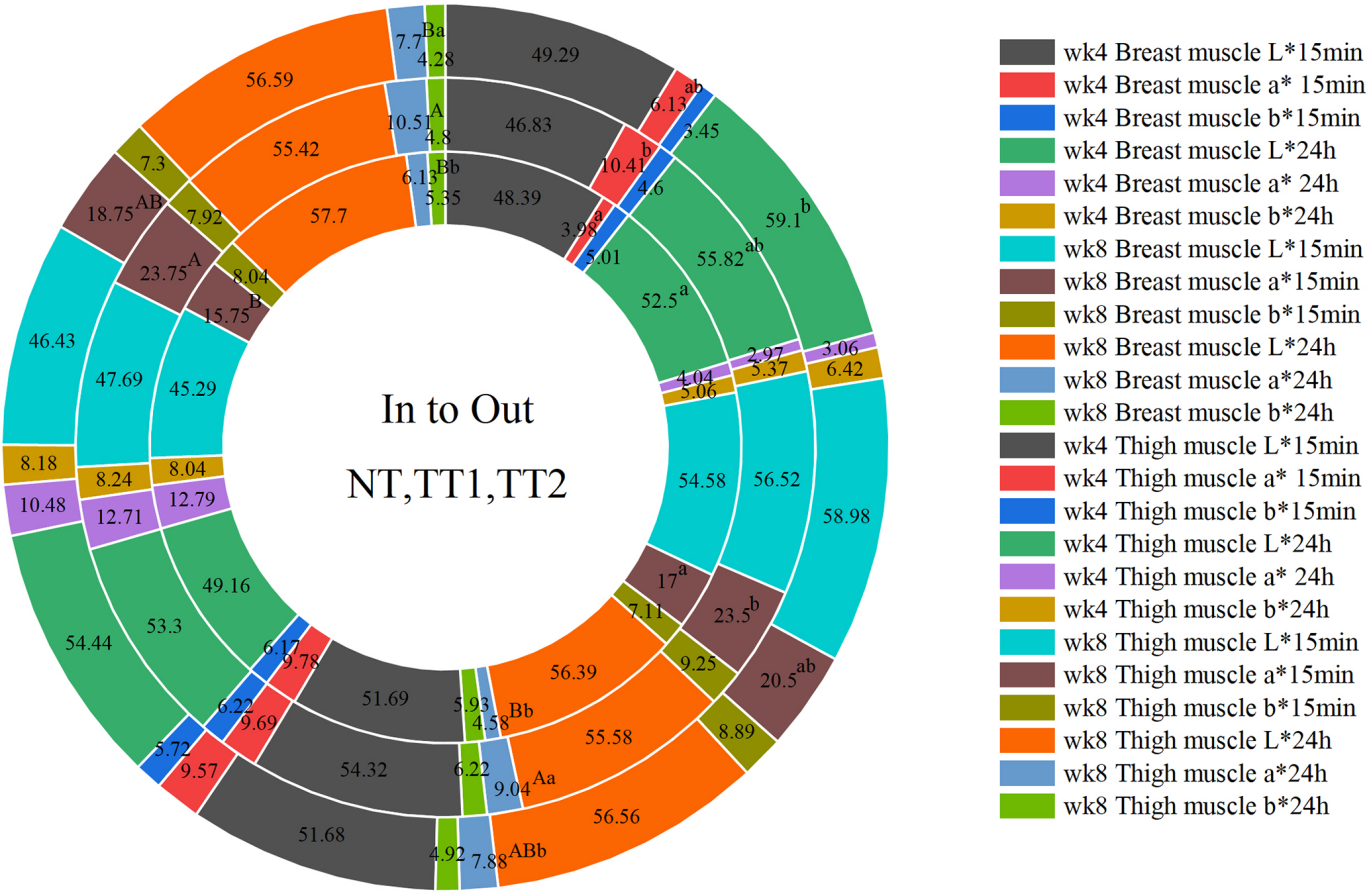
Effects of the replacement of soybean meal with an equivalent amount of fermented feed on the muscle color.

As shown in Fig. 1, at 4 weeks of replacement of soybean meal with an equivalent amount of fermented feed, the TT1 group exhibited a significant increase in the redness ( a* ) of the breast muscle at 15 min ( P < 0.05), and the TT2 group exhibited significant improvement in the brightness ( L* ) of the breast muscle at 24 h ( P < 0.05), with no significant differences in the other indicators ( P > 0.05). At 8 weeks after the replacement of soybean meal with fermented feed, compared with the NT group, the TT1 group presented a significant increase in the redness ( a* ) of the breast and thigh muscles at 15 min and 24 h ( P < 0.05, P < 0.01), but there was no significant difference between the TT2 group and the NT group ( P > 0.05).

**Figure 2:**
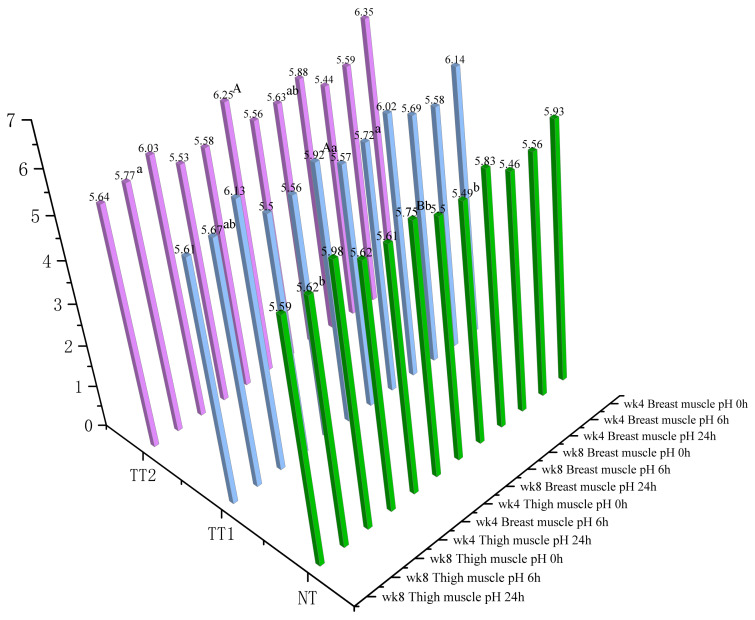
Effects of the replacement of soybean meal with an equivalent amount of fermented feed on the muscle pH.

As shown in Fig. 2, at 4 weeks after the replacement of soybean meal with an equivalent amount fermented feed, the pH of the thigh muscle at 0 h significantly increased ( P < 0.05 or P < 0.01); however, there were no significant differences in the other indicators ( P > 0.05). At 8 weeks after replacement, compared with the NT group, the TT1 group presented a significant increase in the 6 h pH of the breast muscle ( P < 0.05), and the TT2 group presented a significant increase in the 6 h pH of the thigh muscle ( P < 0.05), with no significant differences in the other indicators ( P > 0.05).

### 3.3 Effects of the replacement of different amounts of soybean meal with different amounts of fermented feed on the nutritional composition of muscle

**Figure 3:**
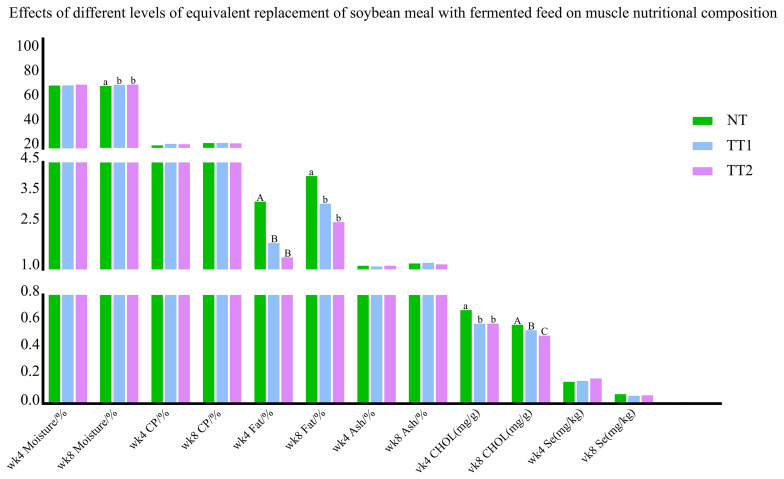
Effects of the replacement of soybean meal with an equivalent amount of fermented feed on the conventional nutritional components of muscle.

As shown in Fig. 3, at 4 and 8 weeks after the replacement of soybean meal with an equivalent amount of fermented feed, there was a significant reduction in fat content ( P < 0.01 and P < 0.05, respectively), a significant decrease in cholesterol content ( P < 0.05 and P < 0.01, respectively), and a significant increase in moisture content ( P < 0.05) in the muscles of laying hens in both the TT1 and TT2 groups; however, there were no significant differences in the other indicators of muscle nutritional composition ( P > 0.05). At 8 weeks, there was a highly significant decrease in cholesterol content in muscle, with an increasing replacement ratio ( P < 0.01).

**Figure 4:**
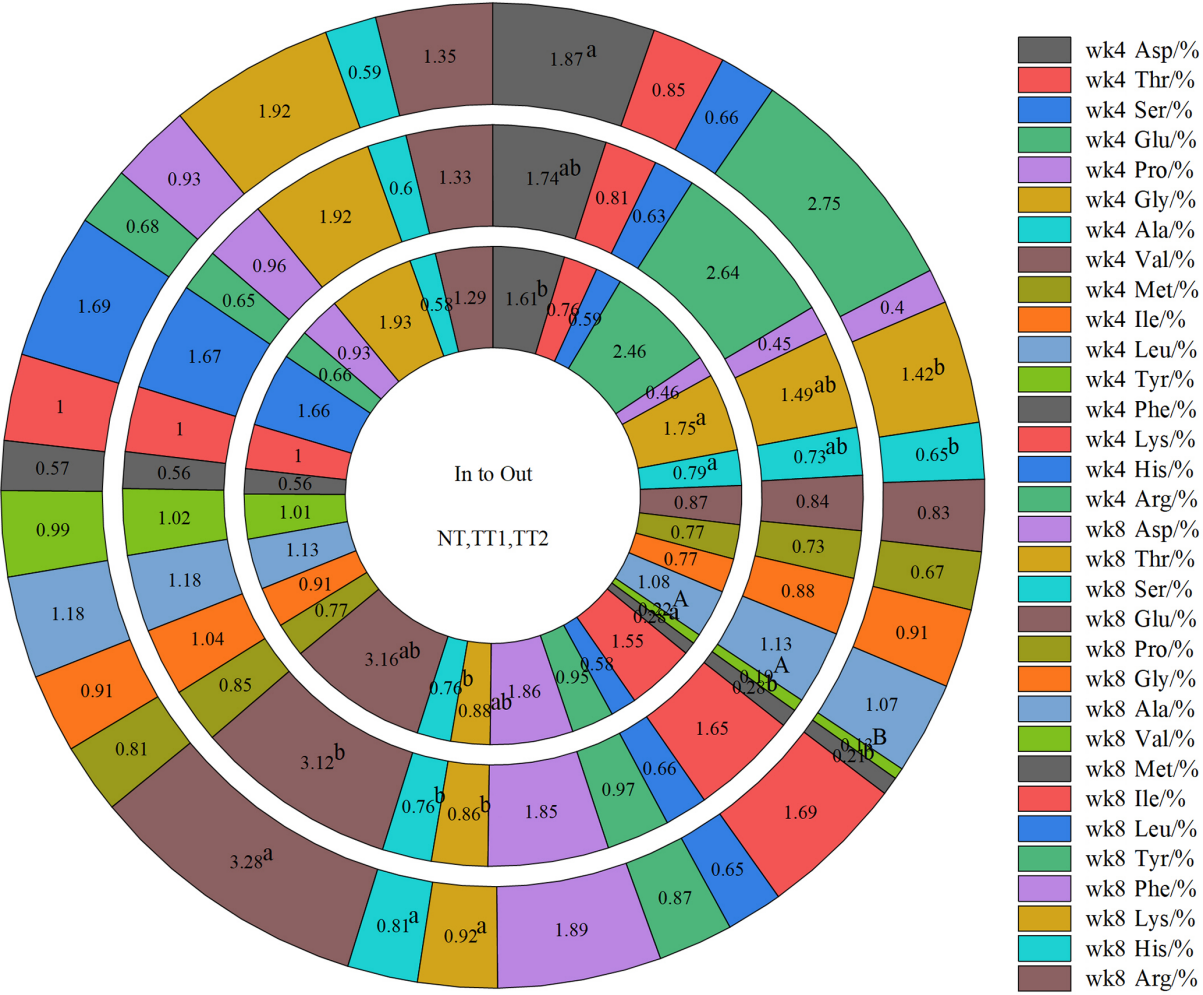
Effects of the replacement of soybean meal with an equivalent amount of fermented feed on the muscle amino acids.

As shown in Fig. 4, at 4 weeks after the replacement of soybean meal with an equivalent amount of fermented feed, the content of 16 amino acids in muscle was not significantly affected in the TT1 group; i.e., the total amounts of these 16 amino acids increased slightly, but the differences were not significant ( P > 0.05). In the TT2 group, there was a significant increase in the aspartic acid content in muscle ( P < 0.05) and a significant decrease in the glycine, alanine, tyrosine, and phenylalanine levels in muscle ( P < 0.01, P < 0.05); however, there was no significant difference in the total amount of 16 amino acids ( P > 0.05). At 8 weeks, the threonine and glutamic acid contents in muscle were significantly greater in the TT2 group than in the TT1 group ( P < 0.05), and the serine content in muscle was significantly greater in the TT2 group than in the NT and TT1 groups ( P < 0.05). There was no significant effect on other indicators ( P > 0.05); the total amount of 16 amino acids increasing slightly, but the difference was not significant ( P > 0.05).

**Figure 5:**
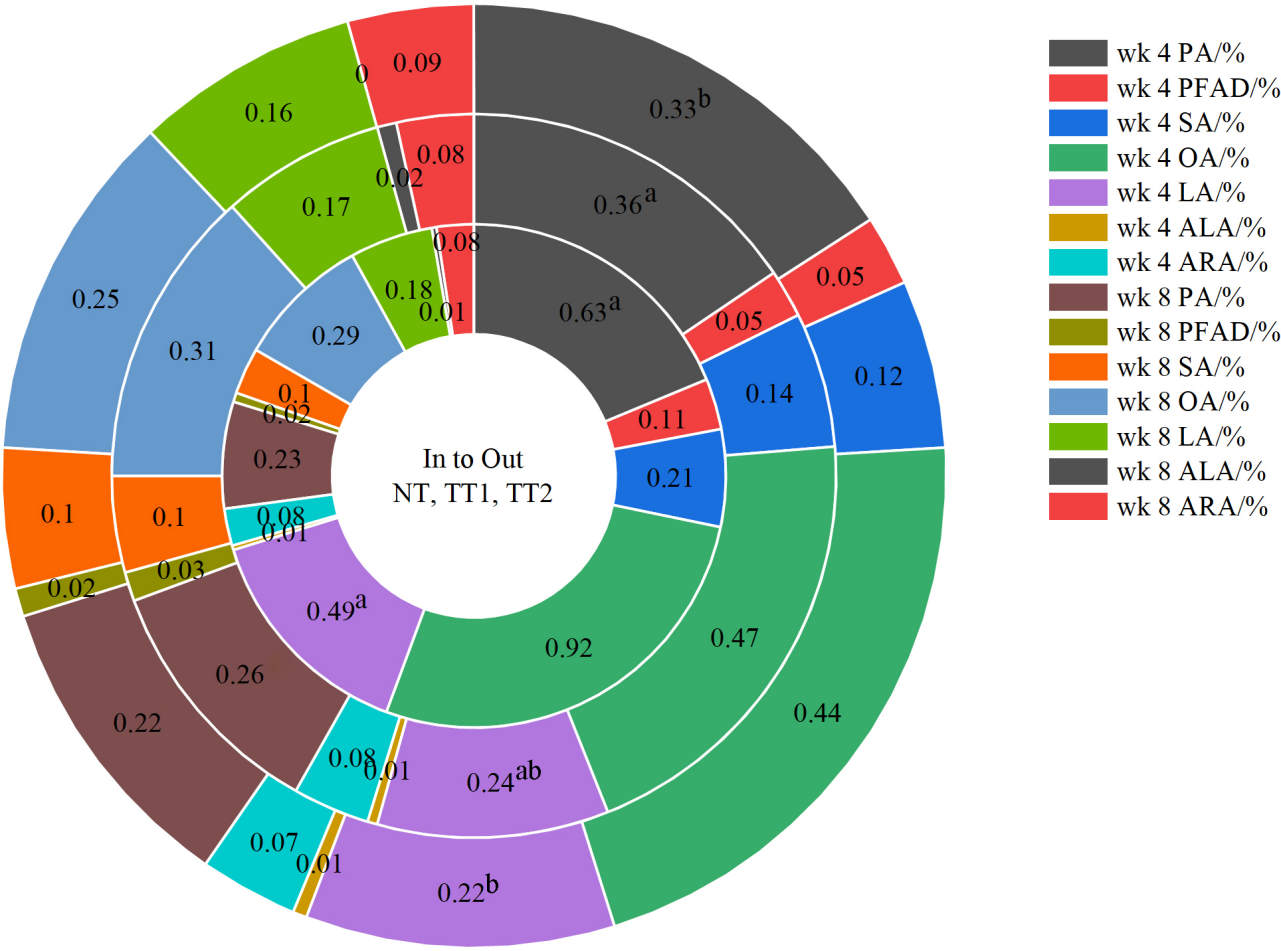
Effects of the replacement of soybean meal with an equivalent amount of fermented feed on the unsaturated fatty acids in muscle.

As shown in Fig. 5, at 4 weeks after the replacement of soybean meal with an equivalent amount of fermented feed, the contents of seven unsaturated fatty acids in the muscle were not significantly different in the TT1 group ( P > 0.05), whereas the contents of palmitic acid and linoleic acid in the muscle significantly decreased in the TT2 group ( P < 0.05). At 8 weeks, the contents of the seven unsaturated fatty acids in muscle were not significantly affected in the TT1 and TT2 groups ( P > 0.05).

## 4 Discussion

Guo et al. (2022) added 20 % fermented feed to the diet of 80-week-old laying hens and reported that the eggshell breakage rate was significantly reduced. Yi et al. (2020) reported that the use of 10 % fermented feed to replace 10 % of the basal diet of Dawu Jinfeng laying hens led to a significant decrease in the abnormal egg rate. Kuang et al. (2023) added 3 % fermented feed to the diet of Hy-Line Brown laying hens and found that the egg breakage rate was significantly reduced. These findings are consistent with the results of this study. Fermented feed not only contains large amounts of probiotics and nutrients but also reduces the content of antinutrients. The addition of fermented feed to the basal diet improved the palatability of feed, increased the nutritional value and utilization of the feed, and enhanced the production performance of the animals, thereby improving egg quality and reducing the egg breakage rate.

Egg weight, the egg shape index, yolk color, eggshell strength, eggshell thickness, and the haugh unit are the main parameters of egg quality (Lu et al., 2023). Egg weight is an important indicator of the economic value of eggs, and it is mainly affected by the breed, age, and diet of laying hens (Han et al., 2023). In this study, at 8 weeks after the replacement of soybean meal with an equivalent amount of fermented feed, the egg weight was significantly greater in the TT2 group than in the NT group. This may be because the probiotics in the fermented feed inhibited pathogenic bacteria in the gastrointestinal tract, thereby improving gastrointestinal tract health and the absorption function of the intestinal villi. As a result, the disease resistance of laying hens was improved, the intestinal digestive function was enhanced, and nutrient utilization was effectively promoted. In studies by Park et al. (2016) and Ye et al. (2017), average egg weight was increased by supplementing the diets of laying hens with fermented plant-based feed, a finding that is consistent with the results of this study.

Yolk color is an important indicator of egg quality, and consumers prefer eggs with a vibrant yolk color (Kowalska et al., 2021). Yolk color can be attributed mainly to the deposition of carotenoids such as lutein, zeaxanthin, and β -carotene in the yolk (Rizzi, 2020). Lu et al. (2023) reported that replacing 4 % or 8 % of soybean meal with fermented feed in the basal diet significantly improved yolk color. The results of the present study revealed that fermented feed significantly improved yolk color, a finding that is consistent with the abovementioned research results. At 4 weeks after the replacement of soybean meal with an equivalent amount of fermented feed, the yolk color was significantly better in the TT2 group than in the NT group. At 4 weeks and 8 weeks after replacement, the yolk color was enhanced due to increased fermented feed replacement, possibly because fermented feed enhances the absorption function of the intestinal villi, leading to increased absorption of zeaxanthin from the feed and, thus, improved yolk color.

Eggshell thickness and strength are the main indicators of eggshell quality and directly affect the egg breakage rate. Improving eggshell quality can reduce the egg breakage rate and increase the economic benefits of layer farming (Zhu et al., 2019b). In this study, At 4 and 8 weeks after the replacement of soybean meal with fermented feed, the eggshell strength and thickness were significantly greater in the TT1 and TT2 groups than in the NT group, with an upward trend for increasing levels of soybean meal replacement. Ribeiro et al. (2014) and Yang et al. (2022a) reported that feeding laying hens with fermented feed enhances eggshell strength and weight and that these values tend to increase with increasing amounts of fermentation additives, a finding that is consistent with the results of this study.

The haugh unit directly reflects the protein quality and freshness of eggs (Liao et al., 2023). In this study, at 4 and 8 weeks after the replacement of soybean meal with an equivalent amount of fermented feed, the haugh units were not significantly different between the TT1, TT2, and NT groups. The results reported by Sanmiguel et al. (2022) are generally consistent with the results of this study.

In this study, a relatively low proportion of fermented feed was used as a substitute for soybean meal. Following an 8-week feeding trial, a notable increase of 3.87 % was observed in the average egg weight. Concurrently, the egg deformity rate decreased by 0.8 %. Moreover, eggshell strength markedly improved, which is anticipated to substantially reduce transportation-related breakage rates. These findings demonstrated that substituting a portion of soybean meal with fermented feed yielded measurable improvements in laying duck performance. Consequently, the utilization of fermented feed as a substitute for soybean meal can, to a certain degree, increase the economic returns associated with laying duck breeding operations.

Conventional meat quality indicators include pH, meat color, water-holding capacity, shear force, cooking loss, and drip loss (Yu et al., 2020). The pH of muscle can serve as a reference indicator of meat freshness and is related to the tenderness, color, and shelf life of meat (Bihan-Duval et al., 2018). In this study, at 8 weeks after the replacement of soybean meal with an equivalent amount of fermented feed, the pH of the breast muscle at 6 h postmortem was significantly higher in the TT1 group than in the NT group; at 4 weeks after the replacement of soybean meal with fermented feed, the pH of the thigh muscle at 0 h postmortem was highly significantly higher in the TT1 group than in the NT group. At 8 weeks after the replacement of soybean meal with fermented feed, the pH of the thigh muscle at 6 h postmortem was significantly higher in the TT2 group than in the NT group, and the pH at other time points in the postmortem period was slightly higher in the TT2 group than in the NT group, but the differences were not significant, indicating that fermented feed can increase the pH of the breast and thigh muscles of laying hens in the late laying period. A study by Guo et al. (2020) revealed that adding fermented feed improved the muscle pH of broiler chickens and enhanced the meat quality of the animals, a finding that is consistent with the results of this study. Meat color is an important indicator for assessing meat quality (Baeza et al., 2022), and it is the most intuitive indicator via which consumers judge meat quality with the naked eye. In general, there is a significant positive correlation between the meat color score and the meat color a* value, with a higher a* value indicating a better sensory quality of meat (Burnett et al., 2020). In this study, at 4 weeks after the replacement of soybean meal with fermented feed, the a* value of the breast muscle was significantly higher in the TT1 group than in the NT group; the a* value of the breast muscle was also higher in the TT2 group than in the NT group, but the difference was not significant. At 8 weeks after the replacement of soybean meal with fermented feed, the a* value of the thigh muscle was significantly greater in the TT1 group than in the NT group, and the a* values of the breast and thigh muscles were greater in the TT2 group than in the NT group, but the differences were not significant. Water-holding capacity refers to the ability of muscle to retain water, which affects the quality and processing of meat. In general, drip loss is used to measure the degree of water-holding capacity of the muscle system. (Woelfel et al., 2002). In this study, at 4 and 8 weeks after the replacement of soybean meal with an equivalent amount of fermented feed, the drip losses of the breast and thigh muscles were significantly lower in the TT1 and TT2 groups compared with the NT group. These findings suggest that fermented feed has a regulatory effect on meat quality in terms of energy, protein, fat, and other nutrient contents.

Approximately 88 %–95 % of the moisture in muscle is located within the cells between actin and myosin filaments. Moisture content has a substantial effect on meat quality; a higher moisture content makes meat juicier, which improves palatability. In this study, at 8 weeks after the replacement of soybean meal with an equivalent amount of fermented feed, the muscle moisture content was significantly higher in the TT1 and TT2 groups than in the NT group, thus improving the palatability of the chicken meat. Fat provides energy for the life activities of animals, but excessive fat deposition leads to a decrease in carcass quality and a waste of feed energy, which is unfavorable for both producers and consumers (Ge et al., 2021). Numerous animal experiments have shown that a high-fat diet is prone to causing a variety of metabolic diseases, such as diabetes, intestinal flora disorders, and obesity (Yang et al., 2022b). Cholesterol is an essential nutrient that is synthesized mainly within the human body and is supplemented in part by dietary sources. However, excessive cholesterol intake can cause dyslipidemia, resulting in diseases such as hyperlipidemia, atherosclerosis, and vascular sclerosis (Xin et al., 2022). Therefore, the nutritional value of meat can be improved by reducing the fat and cholesterol contents in muscle. Research has shown that the fat and cholesterol contents in eggs decrease significantly after 5.0 % bacterial–enzyme cofermented feed is provided. Nie et al. (2020) demonstrated that replacing soybean meal with 6 % cofermented cotton meal in the diet of yellow-feather broiler chickens significantly reduced the abdominal fat content. In a study by Sun et al. (2022), feeding with experimental diets containing 5 %, 10 %, and 15 % fermented feed during the early growth period significantly reduced the muscle cholesterol content of 42 d old broiler chickens. Niu et al. (2021) demonstrated that adding 6 % or 9 % fermented feed to the diet significantly reduced the fat and cholesterol contents of laying hens. In the present study, at 4 and 8 weeks after the replacement of soybean meal with fermented feed, the muscle fat and cholesterol contents were significantly lower in the TT1 and TT2 groups compared with the NT group. The results of this study confirmed that fermented feed is beneficial for improving the muscle quality of (culled) laying hens in the late laying period. This may be because substances such as cellulose produced by the fermentation of soybean meal in fermented feed inhibit the digestion and absorption of triglycerides in the diet by (culled) laying hens in the late laying period, thereby reducing fat deposition and lowering cholesterol levels.

Fatty acids are important determinants of the rich flavor of meat, and a high fatty-acid content improves the nutritional value of meat (Zhang et al., 2019a). Marcincak et al. (2018) reported that adding 10 % fermented feed to broiler feed had a positive effect on the fatty-acid content of chicken breast. Wang et al. (2017) reported that supplementing diets with 3 % and 6 % of fermented cottonseed meal significantly increased the fatty-acid content in muscle – a result that is inconsistent with the findings of the present study; this difference in results may be related to factors such as the type and amount of fermented feed ingredients.

Amino acids are important substances for meat nutrition and crucial components of meat flavor: the higher the content of flavor amino acids in muscle, the more palatable the meat (Chen et al., 2015). Wu et al. (2022) reported that the addition of 1 %–3 % of tea residue–enzyme cofermented feed improved the muscle flavor of Qingjiaoma chickens. Additionally, Li et al. (2021) demonstrated that adding fermented feed to the diet enhances the heat stress resistance and muscle amino acid content of broiler chickens. In the present study, with increasing replacement with fermented feed, the contents of flavor amino acids and total amino acids in the muscle of culled laying hens increased – a finding that is consistent with the results of the above studies. However, there were no significant differences in content among the groups, which may be due to factors such as the low level of equivalent replacement with fermented feed, resulting in nonsignificant differences compared with those in the NT group.

## 5 Conclusion

There was a significant reduction in the abnormal egg rate at 8 weeks after the replacement of 10 % of soybean meal with fermented feed. Egg quality parameters, such as eggshell strength and thickness, significantly improved at 4 and 8 weeks after the equivalent replacement of soybean meal with 5 % or 10 % of an equivalent amount of fermented feed. The best results were observed with a 10 % replacement level after 8 weeks.

Replacement of soybean meal (5 % and 10 %) with fermented feed for 4 and 8 weeks significantly reduced the drip loss of breast muscle and improved the pH and meat quality (e.g., meat color) of the breast and thigh muscles. The best results were observed at a 10 % replacement level for 8 weeks.

At 4 and 8 weeks after the replacement of soybean meal (5 % and 10 %) with an equivalent amount of fermented feed, the fat and cholesterol contents in muscle were significantly reduced, with a greater increase in amino acid levels. The best results were observed at a 10 % replacement level after 8 weeks.

## Data Availability

The data generated or analyzed during this study are provided in full within the published article.

## Statement of novelty

In this study, 70-week-old Hy-Line Sonia laying hens were used as research objects to investigate the effects of replacing soybean meal with an equivalent amount of fermented feed on the egg quality, meat quality, and muscle nutritional composition of late-laying hens, with the goal of providing a theoretical basis for the widespread application of fermented feed in the production of late-laying hens.

## References

[bib1.bib1] Ashayerizadeh A, Dastar B, Shams SM, Sadeghi MA, Zerehdaran S (2017). Fermented rapeseed meal is effective in controlling Salmonella entericaser ovar Typhimurium infection and improving growth performance in broiler chicks. Vet Microbiol.

[bib1.bib2] Baeza E, Guillier L, Petracci M (2022). Review: Production factors affecting poultry carcass and meat quality attributes. Animal.

[bib1.bib3] Burnett DD, Legako JF, Phelps KJ, Gonzalez JM (2020). Biology, strategies, and fresh meat consequences of manipulating the fatty acid composition of meat. J Anim Sci.

[bib1.bib4] Bihan-Duval EL, Hennequet-Antier C, Berri C, Beauclercq SA, Bourin MC, Boulay M, Boitard S (2018). Identification of genomic regions and candidate genes for chicken meat ultimate pH by combined detection of selection signatures and QTL. BMC Genomics.

[bib1.bib5] Chang WY, Yu YH (2022). Effect of Bacillus species–fermented products and essential oils on growth performance, gut morphology, cecal short-chain fatty acid levels, and microbiota community in broilers. Poult Sci.

[bib1.bib6] Chen YC, Yu YH (2022). Bacillus licheniformis-fermented products and enramycin differentially modulate microbiota and antibiotic resistome in the cecaldigesta of broilers. Poult Sci.

[bib1.bib7] Chen ZY, Feng YZ, Cui C, Zhao HF, Zhao MM (2015). Effects of koji-making with mixed strains on physicochemical and sensory properties of Chinese-type soy sauce. J Sci Food Agr.

[bib1.bib8] Darmawan A, Hermana W, Suci DM, Mutia R, Sumiati, Jayanegara A, Ozturk E (2022). Dietary Phytogenic Extracts Favorably Influence Productivity, Egg Quality, Blood Constituents, Antioxidant and Immunological Parameters of Laying Hens: A Meta-Analysis. Animals-Basel.

[bib1.bib9] El-Tarabany MS, Ahmed-Farid OA, El-Bahy SM, Nassan MA, Salah AS (2022). Muscle oxidative stability, fatty acid and amino acid profiles, and carcass traits of broiler chickens in comparison to spent laying hens. Front Vet Sci.

[bib1.bib10] Ge K, Yu DL, Yang R, Zhang RN (2021). Nutrition and feeding strategies in poultry fat regulation. CHINA FEED.

[bib1.bib11] Guo S, Zhang Y, Cheng Q, Xv J, Hou Y, Wu X, Ding B (2020). Partial Substitution of Fermented Soybean Meal for Soybean Meal Influences the Carcass Traits and Meat Quality of Broiler Chickens. Animals-Basel.

[bib1.bib12] Guo L, Lv J, Liu Y, Ma H, Chen B, Hao K, Min Y (2021). Effects of Different Fermented Feeds on Production Performance, Cecal Microorganisms, and Intestinal Immunity of Laying Hens. Animals-Basel.

[bib1.bib13] Guo W, Xu LN, Guo XJ, Wang W, Hao QH, Wang SY, Zhu BC (2022). The impacts of fermented feed on laying performance, egg quality, immune function, intestinal morphology and microbiota of laying hens in the late laying cycle. Animal.

[bib1.bib14] Han GP, Kim DY, Kim KH, Kim JH, Kil DY (2023). Effect of dietary concentrations of metabolizable energy and neutral detergent fiber on productive performance, egg quality, fatty liver incidence, and hepatic fatty acid metabolism in aged laying hens. Poult Sci.

[bib1.bib15] Jazi V, Mohebodini H, Ashayerizadeh A, Shabani A, Barekatain R (2019). Fermented soybean meal ameliorates Salmonella Typhimurium infection in young broiler chickens. Poult Sci.

[bib1.bib16] Krauze M, Cendrowska-Pinkosz M, Matusevicius P, Stepniowska A, Jurczak P, Ognik K (2021). The Effect of Administration of a Phytobiotic Containing Cinnamon Oil and Citric Acid on the Metabolism, Immunity, and Growth Performance of Broiler Chickens. Animals-Basel.

[bib1.bib17] Kuang W, Ji HJ, Wu GS, Fan CY, Yao Y, He ZL, Sun PP (2023). Effects of fermented feed on production performance, egg quality and intestinal flora of laying hens. Animal Husbandry and Veterinary Medicine.

[bib1.bib18] Kowalska E, Kucharska-Gaca J, Kuzniacka J, Lewko L, Gornowicz E, Biesek J, Adamski M (2021). Egg quality depending on the diet with different sources of protein and age of the hens. Sci Rep.

[bib1.bib19] Liao CY, Cui J, Lei JQ, Guo YM, Zhang BK (2023). Effects of Bacillus subtilis Natto NB205 and Its Mutant NBMK308 on Egg Quality in Aging Laying Hens. Life-Basel.

[bib1.bib20] Li R, Chang L, Hou G, Song Z, Fan Z, He X, Hou DX (2019). Colonic Microbiota and Metabolites Response to Different Dietary Protein Sources in a Piglet Model. Front Nutr.

[bib1.bib21] Li XZ, Hao H, Zhou CL, Zhang LW, Zhu Y, Liu Y, Shi YX, Wang FS, Zhang YY (2021). Effects of Compound Microbial Fermented Diets on Growth Performance, Immunity Function and Muscle amino acid content of Broilers. Chinese Journal of Animal Science.

[bib1.bib22] Liu CN, Yang S, Gong Y, Guo YQ, Ye JS, Yang Y (2013). Current Situation and Development Trend of China's Spent Laving Hen Processing Industry. Meat Research.

[bib1.bib23] Liu J, Luo Y, Zhang X, Gao Y, Zhang W (2022). Effects of bioactive peptides derived from cottonseed meal solid-state fermentation on the growth, metabolism, and immunity of yellow-feathered broilers. Anim Sci J.

[bib1.bib24] Lutful Kabir SM (2009). The role of probiotics in the poultry industry. Int J Mol Sci.

[bib1.bib25] Lu ZJ, Zeng N, Jiang SG, Wang XQ, Yan HC, Gao CQ (2023). Dietary replacement of soybean meal by fermented feedstuffs for aged laying hens: effects on laying performance, egg quality, nutrient digestibility, intestinal health, follicle development, and biological parameters in a long-term feeding period. Poult Sci.

[bib1.bib26] Marcincak S, Klempova T, Bartkovsky M, Marcincakova D, Zdolec N, Popelka P, Certik M (2018). Effect of Fungal Solid-State Fermented Product in Broiler Chicken Nutrition on Quality and Safety of Produced Breast Meat. Biomed Res Int.

[bib1.bib27] Missotten JA, Michiels J, Degroote J, De Smet S (2015). Fermented liquid feed for pigs: an ancient technique for the future. J Anim Sci Biotechno.

[bib1.bib28] Ministry of Agriculture and Rural Affairs of the People's Republic of China (2007). Industry Standards of China Determination of livestock and poultry meat quality.

[bib1.bib29] National Health and Family Planning Commission, State Food and Drug Administration (2016). National food safety standard, GB 5009-2016.

[bib1.bib30] National Health and Family Planning Commission, State Food and Drug Administration (2017). National food safety standard Determination of selenium in foods, GB 5009.93-2017.

[bib1.bib31] Nie CX, Wang YQ, Liu YF, Liu JC, Ge WX, Ma X, Zhang WJ (2020). Impacts of Dietary Protein from Fermented Cottonseed Meal on Lipid Metabolism and Metabolomics Profiling in the Serum of Broilers. Curr Protein Pept Sci.

[bib1.bib32] Niu JL, Wei LQ, Luo YQ, Yang WT, Lu QC, Zheng XX, Nie CX (2021). Fermented cottonseed meal improves production performance and reduces fat deposition in broiler chickens. Anim Biosci.

[bib1.bib33] Park JH, Song TH, Kim I (2016). Egg production, egg quality, and cecal microbial populations of layers fed diets supplemented with fermented phytogenic feed additive. Turk J Vet Anim Sci.

[bib1.bib34] Qaisrani SN, van Krimpen MM, Kwakkel RP, Verstegen MW, Hendriks WH (2015). Diet structure, butyric acid, and fermentable carbohydrates influence growth performance, gut morphology, and cecal fermentation characteristics in broilers. Poult Sci.

[bib1.bib35] Rizzi C (2020). Yield Performance, Laying Behaviour Traits and Egg Quality of Purebred and Hybrid Hens Reared under Outdoor Conditions. Animals-Basel.

[bib1.bib36] Ribeiro V, Albino L, Rostagno H, Barreto SL, Hannas M, Harrington D, Araujo FAD, Ferreira HC, Ferreira MD (2014). Effects of the dietary supplementation of Bacillus subtilis levels on performance, egg quality and excreta moisture of layers. Anim Feed Sci Technol.

[bib1.bib37] Sanmiguel RA, Dedousi A, Lopez AD, Sierra DA, Sossidou EN (2022). Effects of feeds, supplemented with humic substances and calcium carbonate, on performance, egg quality and heart rate variability in laying hens. Vet Res Forum.

[bib1.bib38] Sun H, Chen D, Cai H, Chang W, Wang Z, Liu G, Chen Z (2022). Effects of Fermenting the Plant Fraction of a Complete Feed on the Growth Performance, Nutrient Utilization, Antioxidant Functions, Meat Quality, and Intestinal Microbiota of Broilers. Animals-Basel.

[bib1.bib39] Teran LC, Mortera P, Tubio G, Alarcon SH, Blancato VS, Espariz M, Magni C (2022). Genomic analysis revealed conserved acid tolerance mechanisms from native micro-organisms in fermented feed. J Appl Microbiol.

[bib1.bib40] Wang TS, Li J, Ning J, Li Y, Han X, Yin, Huang F (2023). Effects of different processing techniques of palm kernel cake on processing quality of pellet feed, nutrient digestibility, and intestinal microbiota of pigs. J Anim Sci.

[bib1.bib41] Wang YQ, Zhang XY, Liu JC, Nie CX, Zhang FF, Jiang LX, Ma GJ, Zhang WJ (2017). Effects of Cottonseed Meal Fermented by lactobacillus acidophilus on Muscle Nutrients and Flavor Characteristics of Yellow-Feathered Broilers. Chinese Journal of Animal Nutrition.

[bib1.bib42] Woelfel RL, Owens CM, Hirschler EM, Martinez-Dawson R, Sams AR (2002). The characterization and incidence of pale, soft, and exudative broiler meat in a commercial processing plant. Poult Sci.

[bib1.bib43] Wu MJ, Chen XX, Li SY, Bao SS, Zhou XH, Mou LJ, Jiang L, Liu ZS (2022). Effect of Dietary Fermented Feed with Tea Residue, Fungus and Enzyme on Growth Performance Slaughter Performance and Muddle Flavor of Cyan-shank Partridge Chicken. China Poultry.

[bib1.bib44] Xin QW, Zhu ZM, Miao ZW, Hao XN, Li L, Zhang LL, Wu HQ, Wei YB, Zheng NZ (2022). Effects of Marigold Dry Meal on Performance and Egg Quality of Hy-Line Gray Laying Hens. Chinese Journal of Animal Nutrition.

[bib1.bib45] Xu FZ, Zeng XG, Ding XL (2012). Effects of replacing soybean meal with fermented rapeseed meal on performance, serum biochemical variables and intestinal morphology of broilers. Asian-Australas J Anim Sci.

[bib1.bib46] Yang N (2021). Egg production in china: current status and outlook. Front Agr Sci Eng.

[bib1.bib47] Yang X, Sun ZH, Cao CJ, Cheng SZ (2022). Research progress on the fat substitutes in food. Cereals and Oils.

[bib1.bib48] Yang R, Khalid A, Khalid F, Ye M, Li Y, Zhan K, Li Y, Liu W, Wang ZG (2022). Effect of fermented corn by-products on production performance, blood biochemistry, and egg quality indices of laying hens. J Anim Sci.

[bib1.bib49] Ye M, Sun L, Yang R, Wang Z, Qi K (2017). The optimization of fermentation conditions for producing cellulase of Bacillus amyloliquefaciens and its application to goose feed. R Soc Open Sci.

[bib1.bib50] Yi CX, Liu XL, Wang Y, Dong Y (2020). Effects of fermented feed on performance, egg quality and antioxidant capacity of DaWu Golden Phoenix laying hens. Feed industry magazine.

[bib1.bib51] Yu M, Li ZM, Rong T, Wang G, Liu ZC, Chen WD, Li JZ, Li JH, Ma XY (2020). Different dietary starch sources alter the carcass traits, meat quality, and the profile of muscle amino acid and fatty acid in finishing pigs. J Anim Sci Biotechno.

[bib1.bib52] Yu C, Wei J, Yang C, Yang Z, Yang W, Jiang SZ (2018). Effects of star anise (*Illicium verum* Hook.f.) essential oil on laying performance and antioxidant status of laying hens. Poult Sci.

[bib1.bib53] Zhang YF, Zhang JJ, Gong HF, Cui LL, Zhang WC, Ma JW, Chen CY, Ai HS, Xiao SJ, Huang LS, Yang B (2019). Genetic correlation of fatty acid composition with growth, carcass, fat deposition and meat quality traits based on GWAS data in six pig populations. Meat Sci.

[bib1.bib54] Zhu LP, Wang JP, Ding XM, Bai SP, Zeng QF, Su ZW, Xuan Y, Applegate TJ, Zhang KY (2019). The effects of varieties and levels of rapeseed expeller cake on egg production performance, egg quality, nutrient digestibility, and duodenum morphology in laying hens. Poult Sci.

